# Dutasteride, a 5 alpha reductase inhibitor, could be associated with the exacerbation of inflammation in patients with benign prostatic hyperplasia

**DOI:** 10.1111/iju.15612

**Published:** 2024-10-23

**Authors:** So Inamura, Yusuke Fukiage, Hisato Kobayashi, Manami Tsutsumiuchi, Masaya Seki, Minekatsu Taga, Masato Fukushima, Motohiro Kobayashi, Osamu Yokoyama, Naoki Terada

**Affiliations:** ^1^ Department of Urology, Faculty of Medical Sciences University of Fukui Eiheiji Japan; ^2^ Department of Tumor Pathology, Faculty of Medical Sciences University of Fukui Eiheiji Japan

**Keywords:** alpha‐1 blocker, chronic prostatic inflammation, dihydrotestosterone, dutasteride, high endothelial venule‐like vessel, IPSS

## Abstract

**Background:**

α‐1 blockers and dutasteride are widely used as agents to treat benign prostatic hyperplasia (BPH); the impact of these drugs on prostatic inflammation is still unclear. Herein, we investigated the impact of α‐1 blockers and dutasteride treatment of BPH in terms of the degree of prostatic inflammation.

**Materials and Methods:**

Tissue specimens were obtained from 143 BPH patients who were administered α‐1 blockers up until their operation. Thirty‐three of the patients had also been treated with dutasteride before the procedure. The degree of prostatic inflammation was quantified histologically by the ratio of high endothelial venule (HEV)‐like vessels. We divided this retrospective cohort into α‐1 blocker monotherapy and combination therapy (α‐1 blockers + dutasteride) groups and evaluated clinical parameters of the two groups in relation to the degree of chronic prostatic inflammation. At the same time, we assessed factors exacerbating chronic prostatic inflammation.

**Results:**

Comparison of the monotherapy and combination therapy groups showed no significant differences in the parameters of the urodynamic study or degree of chronic prostatic inflammation, whereas the IPSS total score, voiding subscore, nocturia, intermittency, weak stream, and straining were significantly lower in the combination than the monotherapy group. The duration of α‐1 blockers administration was not correlated with the ratio of HEV‐like vessels, while that of dutasteride was strongly correlated (correlation coefficient = 0.595; *p* < 0.001). Multiple regression analysis demonstrated that the duration of dutasteride administration was a key factor exacerbating the degree of chronic prostatic inflammation.

**Conclusions:**

The present study showed that despite their ameliorating effect on prostatic hyperplasia, dutasteride contributed significantly to chronic prostatic inflammation.

**Abbreviations**
5ARI: 5alpha reductase inhibitor
AB: α‐1 blocker
AUR: acute urinary retention
BOO: bladder outlet obstruction
BPH: benign prostatic hyperplasia
DHT: dihydrotestosterone
HEV: high endothelial venule
HoLEP: holmium laser enucleation of the prostate
IPSS: International Prostate Symptom Score
NSAID: nonsteroidal anti‐inflammatory drug
PFS: pressure flow study
TURP: transurethral resection of the prostate

## INTRODUCTION

The progression of benign prostatic hyperplasia (BPH) in men significantly impairs the storage and voiding functions of the lower urinary tract symptoms (LUTS), adversely affecting quality of life. Multiple factors contribute to the progression of BPH, among which dysregulation of the androgen‐signaling cascade and chronic prostatic inflammation are notably pivotal.^1^, ^2^ Chronic prostatic inflammation exacerbates the voiding symptom by increasing prostatic volume and contributing to functional bladder outlet obstruction.^2^, ^3^, ^4^ Chronic prostatic inflammation also affects storage symptom by neural cross‐sensitization to the bladder.^5^

It is hypothesized that urine reflux into the prostatic stroma plays a key role in aggravating prostatic inflammation. Alpha‐1 blockers (ABs) and 5‐alpha reductase inhibitors (5ARIs) have been employed in BPH treatment, with several studies demonstrating their efficacy in mitigating prostatic inflammation.^6^, ^7^ These agents are believed to reduce urine reflux by alleviating bladder outlet obstruction (BOO), thereby suppressing chronic prostatic inflammation.^6^

Testosterone, originating from the testes and adrenal glands, is initially converted to dihydrotestosterone (DHT) by 5‐alpha reductase types 1 and 2. DHT then travels to the prostate, where it activates androgen receptors, leading to the production of signaling factors that regulate prostatic cell growth.^1^ Studies have shown that DHT suppresses inflammation by inhibiting nuclear factor kappa B (NF‐κB), a critical inflammatory process regulator.^8^, ^9^ Additionally, some research has indicated that NF‐κB activation induces cytokines such as interleukin (IL)‐1β, TNF‐α, and IL‐6.^10^, ^11^ In a rat model study, we demonstrated that castration led to increased prostaglandin E2 (PGE2) released from the bladder epithelium and heightened levels of IL‐1β and cyclooxygenase‐2 (COX‐2) in the rat bladder,^12^ suggesting that reduced DHT concentration in males could potentially induce inflammation.

Prescribing 5ARIs is a common approach to halt the progression of BPH, decrease prostate volume, alleviate symptoms, and reduce the risk of acute urinary retention (AUR) and the need for BPH surgery. 5ARIs function by inhibiting 5‐alpha reductase types 1 and 2. Dutasteride, a comprehensive 5ARI targeting both isozymes, effectively reduces DHT concentration in the prostate.^1^ This reduction in DHT concentration might, paradoxically, amplify prostatic inflammation due to DHT's role in inhibiting NF‐κB. We hypothesize that treating BPH patients with dutasteride could exacerbate chronic prostatic inflammation by diminishing the anti‐inflammatory effect of DHT.

Chronic inflammation is characterized as a response to prolonged exposure to detrimental stimuli and as a facilitator of lymphocyte infiltration into inflammation sites.^13^ Lymphocytes migrate to secondary lymphoid organs via high endothelial venules (HEVs), using vascular addressin as a molecular marker.^14^ At inflammation sites, cytokines promote the expression of vascular addressin in vascular endothelial cells, transforming them into HEV‐like structures. Lymphocytes are recruited to these HEV‐like vessels at inflamed sites, which are crucial for chronic inflammation development and serve as a reliable marker of inflammation severity.^4^

This study aims to evaluate the relationship between BPH drug treatments, such as ABs and 5ARIs, and the extent of chronic prostatic inflammation, as assessed by the number of HEV‐like vessels.

## MATERIALS AND METHODS

### Tissue samples and patient clinical information

The study, involving the analysis of human prostate tissues, received approval from the Ethics Committee of the Faculty of Medical Sciences, University of Fukui. We retrieved formalin‐fixed, paraffin‐embedded benign prostatic hyperplasia (BPH) tissues, which were devoid of incidental carcinoma (*n* = 143), from the University of Fukui Hospital's pathological archives. These samples were collected between 2006 and 2016 through transurethral resection of the prostate (TURP) or holmium laser enucleation of the prostate (HoLEP). The decision to perform either TURP or HoLEP was left to the discretion of each attending physician.

We compared two patient groups: one receiving alpha‐blocker (AB) monotherapy (*n* = 110) and the other a combination of AB and dutasteride (*n* = 33). The clinical characteristics of the patients are detailed in Table 1. The mean treatment durations were 26.028 months for the monotherapy group and 23.130 months for the combination therapy group. Patients in the combination therapy group received a daily dose of 0.5 mg dutasteride until the day before surgery. We excluded patients with a history of acute prostatitis, urinary tract infection within 6 months of surgery, or regular use of steroids or nonsteroidal anti‐inflammatory drugs (NSAIDs).

**TABLE 1:** The profiles of patients undergoing ABs monotherapy (*n* = 110) and those receiving combination therapy (*n* = 33).

	Alpha‐1 blockers monotherapy	Combination of alpha‐1 blockers and dutasteride	*p* value
No. of patients	110	33	
Age, years	70.672 ± 7.316	70.424 ± 7.128	0.864
Total prostate volume, mL			
At the initiation of drug therapy	53.350 ± 23.572	63.862 ± 41.157	0.090
Before surgery	55.684 ± 29.976	60.482 ± 21.199	0.394
TZ volume, mL	32.761 ± 25.825	34.175 ± 18.748	0.806
Resected prostate volume, mL	23.182 ± 18.550	27.242 ± 13.942	0.248
Pre‐operative serum PSA levels, ng/mL	5.322 ± 4.754	3.958 ± 2.320	0.127
MECA‐79+/CD34+ vessel ratio	0.046 ± 0.050	0.060 ± 0.077	0.210
Serum CRP levels, mg/dL	0.162 ± 0.271	0.085 ± 0.094	0.135
BMI	22.429 ± 2.886	23.181 ± 2.726	0.202
Existence of AUR, cases/percentage	33 (30.0%)	10 (30.3%)	1.000
IPSS			
Total IPSS score	20.148 ± 7.343	15.130 ± 8.551	0.006
Storage subscores	8.241 ± 3.358	6.792 ± 3.203	0.061
Frequency	3.207 ± 1.541	2.917 ± 1.613	0.420
Urgency	2.402 ± 1.639	2.000 ± 1.642	0.290
Nocturia	2.632 ± 1.202	1.875 ± 1.035	0.006
Voiding subscores	11.862 ± 4.968	8.458 ± 5.748	0.005
Incomplete emptying	2.609 ± 1.580	1.958 ± 1.628	0.079
Intermittency	3.000 ± 1.671	2.208 ± 1.865	0.048
Weak stream	3.862 ± 1.432	2.958 ± 1.655	0.009
Straining	2.391 ± 1.870	1.455 ± 1.654	0.034
QOL score	4.444 ± 1.245	3.913 ± 1.505	0.088
Uroflowmetry			
*Q*_max_, mL/sec	8.997 ± 4.152	8.556 ± 4.205	0.700
*Q*_ave_, mL/sec	4.095 ± 1.796	3.641 ± 2.256	0.443
Voided volume, mL	167.605 ± 103.054	145.975 ± 86.835	0.436
Residual urine volume, mL	105.061 ± 110.599	93.564 ± 99.693	0.719
Pressure flow study			
Parameters for storage function			
FDV, mL	135.464 ± 87.861	126.429 ± 82.750	0.652
SDV, mL	192.804 ± 99.921	188.536 ± 87.331	0.850
Urgency, mL	239.180 ± 117.094	240.520 ± 101.565	0.961
MCC, mL	288.167 ± 132.159	275.481 ± 109.828	0.669
Parameters for voiding function			
Max *P*_det_, cmH_2_O	107.415 ± 34.885	99.407 ± 42.886	0.372
*P*_det_ at *Q*_max_, cmH_2_O	82.154 ± 29.592	82.154 ± 41.107	0.761
BOOI	70.475 ± 30.488	64.354 ± 44.789	0.488
Duration of drug administration			
Alpha 1 blockers (months)	26.028 ± 26.540	23.130 ± 24.423	0.644
Dutasteride (months)	‐	14.767 ± 19.011	‐

*Note*: The patient backgrounds did not significantly differ between the two groups. The subjective IPSS scores for voiding, nocturia, and the total score were significantly lower in the combination therapy group compared to the ABs monotherapy group, while the objective parameters, such as uroflowmetry and pressure flow study, did not differ significantly between the two groups.

Prior to surgery, we assessed the patients' LUTS using the International Prostate Symptom Score (IPSS). This evaluation included scoring three storage symptoms (frequency, urgency, and nocturia) and four voiding symptoms (incomplete emptying, intermittency, weak stream, and straining) on a scale from 0 (not at all) to 5 (almost always), yielding a maximum total score of 35.^2^

The prostate volume was measured by transrectal ultrasound before surgery, and the resected prostate weight was recorded immediately postsurgery. Uroflowmetry assessed presurgical voiding functions by determining maximum flow rate (*Q*_max_), average flow rate (*Q*_ave_), voided volume, and residual urine volume.^15^ Additionally, a pressure flow study (PFS) was conducted for the evaluation of objective lower urinary tract function, following the methods described in our earlier study.^4^

### Immunohistochemistry

We employed the same primary antibodies and methods as in our previous study.^4^ These included MECA‐79 for identifying 6‐sulfo N‐acetyl‐lactosamine on extended core 1 O‐glycans,^16^, ^17^, ^18^ and QBEND10 for recognizing CD34. MECA‐79 is the specific marker of HEV‐like vessels, and we used CD34 as a marker for capillaries, including both MECA‐79 positive and negative. Conventional single immunohistochemistry was performed for both MECA‐79 and CD34, as detailed in a prior reference.^19^

### Assessment of prostatic inflammation

Building on our previous findings, we used the count of HEV‐like vessels as an indicator to quantify chronic prostatic inflammation. We identified three regions with the highest density of MECA‐79+ vessels (“hot spots”) and counted these vessels at ×200 magnification. In parallel, we counted CD34+ vessels in serial sections and calculated the MECA‐79+/CD34+ vessel ratio for each case.

### Statistical analyses

We used the paired Student's *t*‐test to analyze differences between the dutasteride and non‐dutasteride groups. The Chi‐square test examined differences in reported acute urinary retention (AUR) episodes. Pearson's correlation coefficient assessed the relationship between the MECA‐79+/CD34+ vessel ratio and clinical parameters on ordinal scales. All analyses were conducted using SPSS version 22, with significance set at *p* values <0.05.

## RESULTS

### Patients with combination therapy had lower voiding subscores on the preoperative IPSS

We compared each item of the preoperative IPSS between the AB monotherapy group (*n* = 110) and combination therapy group (*n* = 33). Regarding the symptoms related to urinary storage, the score of nocturia was significantly lower in the group receiving combination therapy compared to the group that did not receive dutasteride (monotherapy vs. combination: 2.632 vs. 1.875, *p* = 0.006, Table 1). Other items showed no notable differences between the groups (Table 1).

Regarding the voiding symptoms, all scored items except for incomplete emptying were significantly lower in the combination therapy than the AB monotherapy group (incomplete emptying 2.609 vs. 1.958, *p* = 0.079; intermittency 3.000 vs. 2.208, *p* = 0.048; weak stream 3.862 vs. 2.958, *p* = 0.009; straining 2.391 vs. 1.455, *p* = 0.034; voiding subscore 11.862 vs. 8.458, *p* = 0.005, Table 1). The total IPSS score of the combination group was significantly lower than that of the monotherapy group (20.148 vs. 15.130, *p* = 0.006, Table 1). There was no significant difference in the QOL score between these groups.

Comparisons regarding parameters of uroflowmetry and pressure flow study showed no significant differences in parameters between groups (Table 1).

In the dutasteride combination group, the reasons for adding dutasteride were urinary retention in 9 cases and large prostate volume in 24 cases. Although there was a tendency for the prostate volume at the start of drug therapy to be larger in the combination group compared to the AB monotherapy group, no statistically significant difference was observed (53.350 vs. 63.862 mL, *p* = 0.090, Table 1).

The resected volume was 23.182 mL in the monotherapy group and 27.242 mL in the combination group, with no significant difference between the two groups (*p* = 0.248, Table 1).

### Impact of AB monotherapy versus combination therapy on chronic prostatic inflammation

We assessed the degree of inflammation in BPH by counting HEV‐like vessels and analyzing the variations between the groups treated with AB monotherapy and those receiving combination therapy in cases where data on the duration of administration were available (α1‐blocker: 95 cases, dutasteride: 33 cases). The evaluation showed no significant differences in the level of chronic prostatic inflammation between the monotherapy and combination therapy groups (monotherapy vs. combination; 0.046 vs. 0.060, *p* = 0.210, Table 1).

**FIGURE 1:**
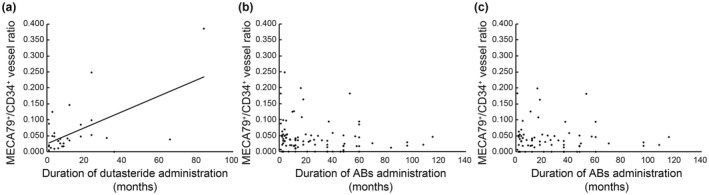
The correlation between the severity of chronic prostatic inflammation and each drug. (a) The length of dutasteride administration demonstrated a positive correlation with the severity of chronic prostatic inflammation. (correlation coefficient = 0.595; *p* < 0.001). (b) Duration of AB administration in all patients (correlation coefficient = −0.145, *p* = 0.081) and (c) The duration of ABs administration in patients receiving monotherapy (correlation coefficient = −0.073, *p* = 0.270) showed no significant correlation with the severity of chronic prostatic inflammation.

We subsequently evaluated the clinical parameters associated with the ratio of HEV‐like vessels. From prior research, we noted that the proportion of HEV‐like vessels was linked to the severity of lower urinary tract symptoms (LUTS) as determined by clinical evaluations.^4^ In patients undergoing dutasteride therapy, our analysis showed a positive correlation between the duration of dutasteride use and the MECA‐79+/CD34+ vessel ratio (correlation coefficient = 0.595; *p* < 0.001, Figure 1a). Conversely, the length of AB therapy showed no correlation with the MECA‐79+/CD34+ vessel ratio, both across all patients (correlation coefficient = −0.145, *p* = 0.081, Figure 1b) and within the AB monotherapy group specifically (correlation coefficient = −0.073, *p* = 0.270, Figure 1c). Additionally, suspected inflammatory factors like obesity and age were not associated with the MECA‐79+/CD34+ vessel ratio in this study (BMI: correlation coefficient = −0.007, *p* = 0.532; Age: correlation coefficient = 0.016, *p* = 0.574). These findings imply that dutasteride treatment might exacerbate chronic prostatic inflammation.

### Relative factors exacerbating chronic prostatic inflammation

We next performed multiple regression analyses on the factors worsening chronic prostatic inflammation. The analysis demonstrated that a long duration of dutasteride administration was a significant factor in exacerbating the degree of chronic prostatic inflammation (*t*‐value = 3.933, *p* < 0.001, Table 2). Thus, longer treatment with dutasteride can be expected to worsen chronic prostatic inflammation.

**TABLE 2:** Factors exacerbating the degree of chronic prostatic inflammation.

	Estimate	Standard error	*t* value	*p* value
MECA79/CD34 vessel ratio	0.135	0.230	0.585	0.566
BMI	−0.002	0.006	−0.299	0.769
BOOI	0.0004	0.0004	1.051	0.307
Duration of dutasteride administration	0.003	0.0007	3.933	<0.001
Age	−0.001	0.003	−0.541	0.595

*Note*: *R* ^2^ = 0.497, adjusted *R* ^2^ = 0.447, *p* = 0.001.

## DISCUSSION

In the present study, we found a positive association between the length of dutasteride treatment and the intensity of chronic prostatic inflammation. This suggests a dosing period‐dependent aggravation of chronic prostatic inflammation by dutasteride. It is notable that our study offers, to our awareness, the first histological evidence highlighting the effects of dutasteride on prostatic inflammation.

5ARIs play a critical role in testosterone metabolism, which is pivotal in understanding their effects on chronic prostatic inflammation observed in our study. It is well established that 5ARIs significantly reduce dihydrotestosterone (DHT) levels by inhibiting the 5‐alpha reductase. The interaction between DHT, which has a higher affinity for the androgen receptor (AR) than testosterone,^20^ and AR leads to several physiological effects, including anti‐inflammatory effects.^8^, ^21^ This is particularly relevant as DHT's higher affinity for AR makes it a more potent mediator in reducing proinflammatory cytokines such as IL‐1 and tumor necrosis factor‐alpha (TNF‐α) through AR‐dependent inhibition of the NF‐κB pathway. There are reports that NF‐kappa B is involved in the formation of high endothelial venules (HEVs),^22^, ^23^ so it is possible that androgens may also influence the formation of HEVs through NF‐kappa B.

It is also important to consider the change in the level of male hormones during the treatment by 5ARIs. Significantly, Amory et al. noted that while DHT levels decrease markedly post‐5ARI treatment, testosterone levels show a modest increase.^24^ Given the critical role of DHT in mediating anti‐inflammatory effects through AR, a reduction in DHT could potentially exacerbate prostatic inflammation, despite the slight increase in testosterone. Therefore, our findings support the hypothesis that prolonged dutasteride treatment correlates with increased chronic prostatic inflammation, likely due to diminished DHT levels.

Urine reflux into the prostatic duct and stroma was associated with the occurrence of chronic inflammation.^25^ BOO is one of the reasons for urine reflux in the prostate, and relieving BOO contributes to arresting the progression of chronic prostatic inflammation.^26^ ABs and 5ARIs are both major agents for alleviating BOO in patients with BPH; however, these two drugs suppress BOO in different ways. ABs relieve the degree of BOO via relaxation of prostatic smooth muscle cells, whereas 5ARIs relieve the BOO by decreasing the prostatic volume.^26^ As a result, the amount of urine reflux into the prostatic stroma might decrease, and it is possible that these drugs would suppress chronic prostatic inflammation by these mechanisms.^6^, ^26^

However, our present study showed no relationship between ABs and chronic prostatic inflammation and even a positive correlation between duration of dutasteride treatment and chronic prostatic inflammation. The results suggested that since the cases examined in this study were only those patients who required surgery due to persistent BOO despite medication, the expected reduction in lower urinary tract obstruction due to volume reduction from dutasteride or functional obstruction relief from ABs therapy did not fully manifest. Consequently, the inflammation‐promoting effect of dutasteride due to the suppression of DHT production became more prominent.

Another possible reason that 5ARIs would enhance prostatic inflammation is the prostatic ischemia due to administration of 5ARIs. A previous study using the human BPH tissue obtained from TURP demonstrated that the density of arteries and vessels in the prostate was lower in the patients who received dutasteride preoperatively than that in the patients who did not.^27^ Lower density of blood vessels would lead to prostatic ischemia. Nomiya and Kobayashi independently reported that prostatic ischemia promoted the secretion of inflammatory cytokines in animal studies.^28^, ^29^ Thus, the 5ARIs administration would lead to prostatic inflammation by reducing blood flow in the prostate. However, further studies are required to prove these hypotheses.

In this study, while a positive correlation was observed between the duration of dutasteride administration and the severity of inflammation in the dutasteride‐treated group, there was no significant difference in the severity of inflammation when comparing the AB monotherapy group with the dutasteride combination therapy group. Possible reasons for this result include the difference in the number of cases between the two groups, especially the number of dutasteride group was quite smaller than that of monotherapy group, the likelihood that many patients are influenced by factors other than DHT that affect prostate inflammation, and the retrospective nature of this study, which did not allow for sufficient standardization of patient backgrounds. Further investigation is needed to understand these results more thoroughly.

The benefit of treatment with 5ARIs for BPH has been established in many past studies, despite its potential negative impact on inflammation. Our findings also demonstrated that the patients treated by combination therapy with ABs and dutasteride had lower scores in IPSS total score, voiding subscore, intermittency, weak stream, straining, and nocturia than those by ABs monotherapy. The reason for the better IPSS scores in the combination group could be that the prostate volume slightly decreased between the start of drug therapy and surgery. This reduction, likely due to the add‐on effect of dutasteride, may have alleviated mechanical obstruction, leading to an improvement in the voiding subscore. Based on this study, it can be said that the impact of dutasteride‐induced prostatic inflammation was limited to clinical situations. We hypothesized that chronic prostatic inflammation would indeed adversely affect voiding and storage dysfunction; however, in most cases, the effect of reducing the prostatic volume by 5ARIs might exceed the enhancement of prostatic inflammation. From this point of view, we speculated that suppression of inflammation during the treatment with 5ARIs might amplify the effects of 5ARIs. Di Silverio et al. reported that their BPH patients who received combination therapy with finasteride (a 5‐alpha reductase type 1 inhibitor) and rofecoxib (a COX‐2 inhibitor) had improved IPSS scores and earlier uroflowmetry *Q*_max_ values compared to patients treated with finasteride monotherapy.^30^ This report supports the usefulness of combination treatment with 5ARIs and anti‐inflammatory agents. Thus, we believe that the control of inflammation would bring out more of the potential of 5ARIs. However, we should pursue the precise mechanisms and the benefits of 5ARIs for BPH regarding inflammation by animal examinations and prospective clinical trials in the future. Furthermore, the finding that the use of drugs affecting DHT production might influence the degree of prostate inflammation provides new insights that enhance our understanding of the pathophysiology of benign prostatic hyperplasia.

The present study includes several limitations. (1) this is a retrospective study; (2) the sample size was small and the difference in the number of samples between the two groups; (3) the cohort of this study almost consists of severe BPH patients; (4) lack of uniformity in surgical techniques; (5) in some cases, the tissue sample specimens are old; (6) because the histologic characteristics of most cases in the study cohort were classified as fibromuscular dominant, the cases with glandular dominant were not examined in this study. In order to assess the impact of ARIs more accurately on prostate inflammation, inclusion of a broad range of patients with mild to severe BPH is necessary.

Our retrospective study revealed that the duration of dutasteride treatment was positively correlated with the magnitude of prostatic inflammation in patients with BPH. It suggested that dutasteride might enhance the chronic prostatic inflammation. However, due to the small number of cases, further investigation is needed to clarify the relationship between dutasteride and inflammation. At the same time, dutasteride improved the voiding dysfunction of the patients. It might be possible to further improve the positive effects of dutasteride by suppressing inflammation.

## AUTHOR CONTRIBUTIONS

**So Inamura:** Conceptualization; investigation; methodology; writing – review and editing; writing – original draft; resources; project administration; formal analysis; visualization. **Yusuke Fukiage:** Methodology; software; data curation. **Hisato Kobayashi:** Methodology; validation. **Manami Tsutsumiuchi:** Methodology. **Masaya Seki:** Methodology. **Minekatsu Taga:** Methodology. **Masato Fukushima:** Methodology. **Motohiro Kobayashi:** Supervision. **Osamu Yokoyama:** Supervision. **Naoki Terada:** Supervision; funding acquisition.

## CONFLICT OF INTEREST STATEMENT

Naoki Terada is an Editorial Board member of International Journal of Urology and a co‐author of this article. To minimize bias, they were excluded from all editorial decision‐making related to the acceptance of this article for publication. The authors except for Naoki Terada declare no conflict of interest.

## APPROVAL OF THE RESEARCH PROTOCOL BY AN INSTITUTIONAL REVIEWER BOARD

The protocol for this research project has been approved by a suitably constituted Ethics Committee of the institution and it conforms to the provisions of the Declaration of Helsinki. Committee of the University of Fukui, Approval No. 26‐62.

## INFORMED CONSENT

All informed consent was obtained from the patients.

## REGISTRY AND THE REGISTRATION NO. OF THE STUDY/TRIAL

N/A.

## ANIMAL STUDIES

N/A.
